# Octreotide, a Somatostatin Analogue, Fails to Inhibit Hypoxia-induced Retinal Neovascularization in the Neonatal Rat

**DOI:** 10.1155/EDR.2000.39

**Published:** 2000

**Authors:** Edward Averbukh, Michael Halpert, Ravit Yanko, Lutza Yanko, Jacob Peèr, Samuel Levinger, Allan Flyvbjerg, Itamar Raz

**Affiliations:** 1 Departments of Ophthalmology Hadassah University Hospital P. O. Box 12000 Jerusalem 91120 Israel; 2 Internal Medicine Hadassah University Hospital Jerusalem Israel; 3 Department of Diabetes and Endocrinology Institute of Experimental Clinical Research Aarhus Kommunehospital Denmark

## Abstract

*Objective:* Octreotide, a somatostatin analogue, has been shown to prevent angiogenesis in diverse *in vitro* models. We evaluated its effect on retinal neovascularization *in vivo*, using a neonatal rat retinopathy model.

*Methods:* We used, on alternating days, hypoxia (10% O_2_) and hyperoxia (50% O_2_) during the first 14 days of neonatal rats, to induce retinal neovascularization. Half of the rats were injected subcutaneously with octreotide 0.7 μg/g BW twice daily. At day 18 the eyes were evaluated for the presence of epiretinal and vitreal hemorrhage, neovascularization and epiretinal proliferation. Octreotide pharmacokinetics and its effect on serum growth hormone (GH) and insulin-like growth factor I (IGF-I) were examined in 28 rats.

*Results:* Serum octreotide levels were 667 μg/1 two hours after injection, 26.4 μg/1 after nine hours and 3.2 μg/1 after 14 hours. GH levels were decreased by 40% (*p* = 0.002) two hours after injection but thereafter returned to baseline. IGF-I levels were unchanged two hours after injection and were elevated by 26% 14 hours after injection (*p* = 0.02). Epiretinal membranes were highly associated with epiretinal hemorrhages (*p* < 0.001), while retinal neovascularization was notably associated with vitreal hemorrhages (*p* <  0.001).

*Conclusions:* Twice-daily injections of octreotide failed to produce sustained decrease in serum GH, but produced rebound elevation of serum IGF-I. Accordingly, no statistically significant effect of injections on retinal pathology was noted. This finding, however, does not contradict our assumption that GH suppression may decrease the severity of retinopathy.


*Int. Jnl. Experimental Diab. Res.*, Vol. 1, pp. 39–47
Reprints available directly from the publisher
Photocopying permitted by license only

© 2000 OPA (Overseas Publishers Association) N.V.
Published by license under
the Harwood Academic Publishers imprint,
part of The Gordon and Breach Publishing Group.
Printed in Malaysia.

### Octreotide, a Somatostatin Analogue, Fails to Inhibit Hypoxia-induced Retinal Neovascularization in the Neonatal Rat

EDWARD AVERBUKH[a], MICHAEL HALPERT[a], RAVIT YANKO[b], LUTZA YANKO[a], JACOB PEÈR[a], SAMUEL LEVINGER[a], ALLAN FLYVBJERG[c] and ITAMAR RAZ[b,*]

[a] *Departments of Ophthalmology,* [b] *Internal Medicine, Hadassah University Hospital, Jerusalem, Israel;* [c] *Department of Diabetes and Endocrinology, Institute of Experimental Clinical Research, Aarhus Kommunehospital, Denmark*

*(Received 10 June 1999; In final form 6 October 1999)*

***Objective:*** **Octreotide, a somatostatin analogue, has been shown to prevent angiogenesis in diverse *in vitro* models. We evaluated its effect on retinal neovascularization *in vivo*, using a neonatal rat retinopathy model.**
***Methods:*** **We used, on alternating days, hypoxia (10%$O_2$) and hyperoxia (50%$O_2$) during the first 14 days of neonatal rats, to induce retinal neovascularization. Half of the rats were injected subcutaneously with octreotide 0.7 μg/g BW twice daily. At day 18 the eyes were evaluated for the presence of epiretinal and vitreal hemorrhage, neovascularization and epiretinal proliferation. Octreotide pharmacokinetics and its effect on serum growth hormone (GH) and insulin-like growth factor I (IGF-I) were examined in 28 rats.**
***Results:*** **Serum octreotide levels were 667 μg/l two hours after injection, 26.4 μg/l after nine hours and 3.2 μg/l after 14 hours. GH levels were decreased by 40% ($p = 0.002$) two hours after injection but thereafter returned to baseline. IGF-I levels were unchanged two hours after injection and were elevated by 26% 14 hours after injection ($p = 0.02$). Epiretinal membranes were highly associated with epiretinal hemorrhages ($p < 0.001$), while retinal neovascularization was notably associated with vitreal hemorrhages ($p < 0.001$).**
***Conclusions:*** **Twice-daily injections of octreotide failed to produce sustained decrease in serum GH, but produced rebound elevation of serum IGF-I. Accordingly, no statistically significant effect of injections on retinal pathology was noted. This finding, however, does not contradict our assumption that GH suppression may decrease the severity of retinopathy.**

*Keywords:* Retinopathy of prematurity, angiogenesis, insulin-like growth factor, growth hormone, neovascularization

### INTRODUCTION

Retinal neovascularization is a common feature of several eye diseases characterized by retinal hypoxia, such as proliferative diabetic retinopathy (PDR) and retinopathy of prematurity (ROP). While the underlying pathology may be different in these conditions, the neovascular process itself leads to destruction of normal retina and eventually causes blindness.

*Corresponding author. Department of Ophthalmology, Hadassah University Hospital, P. O. Box 12000, Jerusalem 91120, Israel. Tel.: +972-2-677 6927, Fax: +972-2-643 4434, e-mail: edward@md2.huji.ac.il

Growth hormone (GH) and insulin-like growth factor I (IGF-I) have been implicated in the pathogenesis of PDR (Lundbaek *et al.*, 1970; Dills *et al.*, 1991). IGF-I has been shown to have angiogenic properties in animal models (Grant *et al.*, 1993), and serum IGF-I levels have been reported to be elevated in patients with PDR (Merimee, Zapf and Froesch, 1983). High vitreous levels of IGF-I and IGF binding proteins (IGFBPs) were found in patients with neovascular eye disease related to diabetes and to other ischemic retinal conditions (Meyer-Schwickerath *et al.*, 1993). In addition, a recent study demonstrated the essential role of GH and IGF-I in the development of retinal neovascularization in the ROP model in mice (Smith *et al.*, 1997).

Decades ago, before the advent of lasers, elimination of GH production by pituitary ablation was an accepted therapy for PDR (Bradley, Rees and Fager, 1965). This approach was abandoned later because of obvious drawbacks, although several studies showed its effectiveness (Kohner *et al.*, 1972). Pharmacological inhibition of the GH secretion was shown to be an effective substitute for hypophysectomy in treatment of clinical PDR (McCombe *et al.*, 1991) and experimental ROP (Smith *et al.*, 1997).

Octreotide, a long-acting somatostatin analogue, is a potent inhibitor of GH secretion with a plasma half-life of 90 minutes (Bauer *et al.*, 1982). Octreotide can be used to lower GH and IGF-I levels (Plewe *et al.*, 1987). In addition, octreotide has been shown to have direct anti-angiogenic properties in human retinal endothelial cells culture (Grant, Caballero and Millard, 1993) and in the vascular growth model of the chick-embryo chorioallantoic membrane (Woltering *et al.*, 1991). Octreotide was tested in human clinical trials for treatment of both early (Kierkegaard *et al.*, 1990) and severe PDR (Mallet *et al.*, 1992; Lee *et al.*, 1988). Though several positive trends were shown in these studies, the small size of the study groups and lack of adequate controls do not allow us to draw results. We studied the impact of systemic octreotide treatment on retinal neovascularization in a neonatal rat ROP model in which we demonstrated a possible role of the retinal IGF system in ischemia-induced neovascularization (Averbukh *et al.*, 1998).

### METHODS

### Animals

Fifty-six neonatal Sabra rats (provided by the Harlan Laboratories, Jerusalem, Israel) were divided into experimental groups of eight to ten animals from mixed litters, not divided according to gender. At the age of one day the group with the lactating mother were placed in an incubator with a controlled oxygen concentration. The lactating rats had free access to standard rat chow and tap water. All the animals were maintained and treated in accordance with the Association for Research in Vision and Ophthalmology statement on the use of animals in ophthalmic and vision research. Retinal neovascularization was induced by alternating oxygen concentrations as previously described (Reynaud and Dorey, 1994; Penn, Tolman and Lowery, 1993). Briefly, the animals were exposed on alternating days to hypoxia (10% $O_2$ – 24 h) and hyperoxia (50% $O_2$ – 24 h) during their first 14 days, followed by four days in room air. Hypoxia was produced by mixing air with nitrogen, and hyperoxia by mixing air with oxygen. A minimal flow of two liters per minute was maintained. Half of the rats were injected subcutaneously with octreotide 0.7 μg/g BW twice daily, while the other half were injected with saline during the whole period. At day 18 the rats were anesthetized with ether and sacrificed. The eyes were enucleated and placed in formaldehyde. To evaluate the effect of octreotide injection on GH and IGF-I levels, we checked the serum levels in another 28 animals on days 7 (11 animals) and 14 (17 animals).

### Evaluation of Neovascularization-associated Changes

In the neonatal ROP model, high oxygen levels impede the normal vascularization process (Chang-Ling *et al.*, 1992) and induce vaso-obliteration (Pierce, Foley and Smith, 1996), thus creating avascular areas in the periphery of the retina. These areas are prone to ischemia when the oxygen level is lowered. Severe hypoxia is also associated with pre-retinal and vitreal hemorrhages in neonatal rats. We observed severe intraocular hemorrhages in neonatal rats after 24 hours' exposure to hypoxia, suggesting that these hemorrhages are primary phenomena and not secondary to neovascularization (unpublished data). Blood on the retina, as well as ischemia-associated factors, may cause epiretinal proliferation and neovascularization which are typical for many retinopathies, such as ROP and diabetic retinopathy. These morphologic changes were the subject of our study when comparing the treated animals with the controls.

Each formalin-fixed, paraffin-embedded eyeball was examined for existence of gross pathology. Then it was sliced using coronal cuts starting from the optic nerve and ending close to the limbus. If intraocular hemorrhages were noted, cuts were made through them. Otherwise, ten representative histological slides were prepared skipping approximately equal numbers of cuts in between. The slides were stained with hematoxylin-eosin and examined and scored in a masked fashion by one examiner. Each eye was assessed for various manifestations of retinal disease. We looked for epiretinal hemorrhages (RH) (defined as blood trapped between the retina and the posterior hyaloid face), vitreal hemorrhages (VH) (blood cells dispersed in the vitreous), epiretinal proliferation (proliferative vitreoretinopathy, PVR) and neovascularization (NV). We defined our findings as NV only when we saw discrete blood vessels on the inner side of the internal limiting membrane (Fig. 1A), while the finding of fibrovascular proliferation and membrane formation inside the vitreous cavity (Fig. 1B) was defined by us as PVR. This separation of the proliferative changes into NV and PVR was dictated by our suspicion that we are seeing two separate phenomena with different pathogenesis.

Each manifestation was given a score between 0 (non-existent) and 3 (severe) according to the number of slides containing the specific pathology and the subjective perception of the severity of pathology. For example, if neovascularization was seen only on one slide and was of limited extent it was assigned a score of one. The statistical significance of the difference between the scores was evaluated using the Mann-Whitney Rank Sum Test. Correlation between RH and VH on the one hand and PVR and NV on the other hand was evaluated using the Spearman Rank Order Correlation test. All statistical analysis was performed using SigmaStat for Windows software (Jandel Corporation, San Rafael, CA).

### Serum Octreotide, GH and IGF-I Levels

Serum octreotide, GH and IGF-I blood levels were evaluated 2 hours after subcutaneous injection of 10 µg octreotide in five 7-day-old animals with an average weight of 15 g (0.7 µg/g BW). Five control animals of the same age and weight were injected with saline. Similarly, the levels were measured in seventeen 14-day-old animals with an average weight of 30 g after injection of 20 µg of octreotide. Five animals were sacrificed 9 hours after injection, and four animals 14 hours after injection. Eight controls of similar weight and age were injected with saline. Average levels of octreotide, GH and IGF-I were calculated for each group. We used a one-tail t-test for statistical analysis.

Serum rat growth hormone (rGH) was measured by radioimmunoassay (RIA) using a specific polyclonal rabbit rGH antibody and rGH as standard. The ingredients for the RIA were obtained from Amersham (Amersham International, Amersham, Bucks, UK). Intra- and

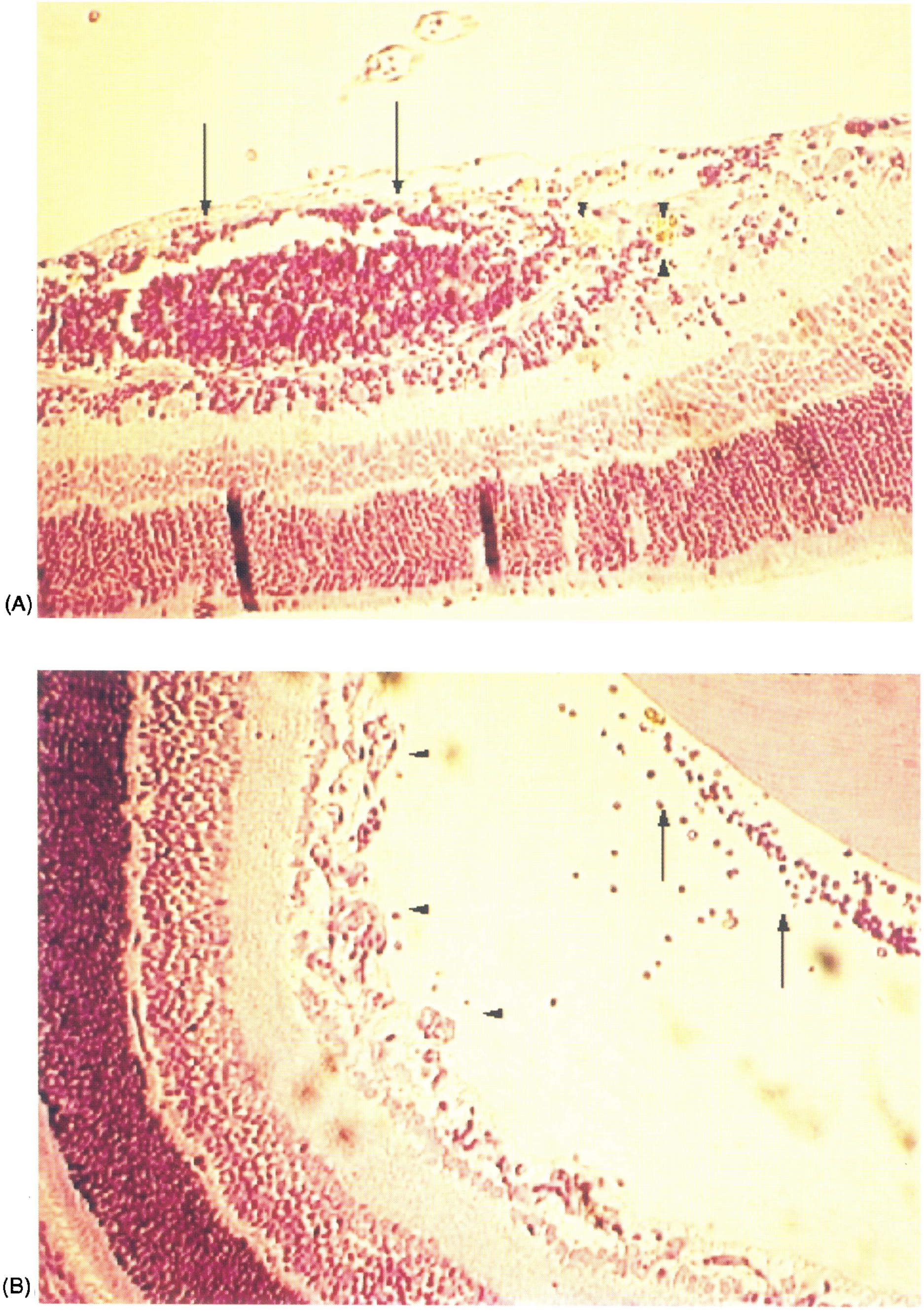

FIGURE 1 Typical findings in the affected eyes. Slide A illustrates big epiretinal hemorrhage (arrows) with epiretinal membrane formation around it. Some hemosiderin spots (arrow heads) are seen inside the hemorrhage, indicating the long-standing nature of the bleeding. Slide B shows vitreous hemorrhage (arrows) with retinal neovascularization (arrow heads). Hematoxylin-eosin stain, magnification × 10. (See Color Plate I).

interassay coefficients of variations were less than 5% and 10% respectively. Serum IGF-I was measured after extraction with acid-methanol (30 µl serum and 750 µl acid methanol) (Furlanetto and Marino, 1987). The mixture was incubated for two hours at room temperature, centrifuged, and 25 μl of the supernatant was diluted 1:200 before analysis. Serum IGF-I was measured by RIA using a polyclonal rabbit antibody (Nichols Institute Diagnostics, San Juan Capistrano, Calif., USA) and recombinant human IGF-I as standard (Amersham International, Amersham, Bucks, UK). Mono-idionated IGF-I $^{125}$I-($^{31}$Tyr)-IGF-I was obtained from Novo-Nordisk A/S Bagsvaerd, Denmark. When exposing the serum extract to western ligand blotting (WLB), no IGFBPs could be identified and furthermore, semilog linearity of biosynthetic IGF-I and serum extracts was seen, indicating antigen similarity and the fact that no IGFBPs interfered in the RIA. Intra- and interassay coefficients of variations were below 5 and 10% respectively.

Serum octreotide was measured by RIA as previously described (Ørskov, Thomsen and Yde, 1968). Intra- and interassay coefficients of variations were less than 5% and 10% for all assays.

### RESULTS

Fifty-two eyes of 26 animals were examined in the treatment group and 59 eyes of 30 animals in the control group (one eye was not suitable for histologic preparation).

### Serum Octreotide, GH and IGF-I Levels

Octreotide levels were high two hours after injection, but much lower later (Tab. I). To calculate the biological half-life time of octreotide in our experiment we compared the octreotide level two hours after injection with that nine hours after injection. The serum level was reduced from 667.0 μg/l to 26.4 μg/l in these seven hours (420 minutes), which is less than five [$\log_2(667.0/26.4) = 4.66$] half-lives, thus, a half-life time of approximately 90 minutes (420 min/4.66 = 90.1 min.). Similarly, basing the calculation of octreotide half-life time on the difference between the octreotide level at 9 hours and its level at 14 hours results in a half-life time of 98 minutes.

Serum GH levels were decreased by 40% ($p = 0.002$) two hours after injection of octreotide as compared with saline injected animals (Tab. I). However, 9 and 14 hours after injection, GH level was comparable in the octreotide and the control groups. Serum IGF-I level was similar in the treated and the control groups two hours after injection, but was elevated in the treated group 9 and 14 hours after injection (by 19% and 26%, $p = 0.06$ and $p = 0.02$ respectively).

### Histologic Findings

The scores of histologic findings in the eyes are summarized in Table II. Epiretinal and vitreal hemorrhages were the most frequently observed findings in both the octreotide-treated and the control groups with approximately 45% of the eyes affected by each one of them and 70% affected by any of them. PVR was less common, affecting less than 40% of the eyes in each group. NV was found only in 35% of the octreotide-treated and in 27% of the control

TABLE I Octreotide, GH and IGF-I levels after injection of octreotide

| Time after injection | $n$ | Age (days) | Octreotide μg/l | GH μg/l | GH % of control | IGF-I μg/l | IGF-I % of control |
|---|---|---|---|---|---|---|---|
| Control | 6 | 7 | 0 | 73.7 ± 8.7 | 100 ± 12 | 66.8 ± 7.6 | 100 ± 11 |
| 2 hours | 5 | 7 | 667.0 ± 42.5* | 44.6 ± 8.4† | 60 ± 11 | 66.4 ± 9.0 | 99 ± 13 |
| Control | 8 | 14 | 0 | 49.5 ± 19.5 | 100 ± 39 | 77.6 ± 8.9 | 100 ± 11 |
| 9 hours | 5 | 14 | 26.4 ± 5.1 | 48.3 ± 11.2 | 98 ± 27 | 92.4 ± 14.2 | 119 ± 18 |
| 14 hours | 4 | 14 | 3.2 ± 0.7 | 41.3 ± 18.1 | 83 ± 37 | 98.0 ± 13.1‡ | 126 ± 17 |

*Values are given as mean ± SEM; †$p = 0.002$; ‡$p = 0.02$.

TABLE II Scoring of findings in the eyes of the octreotide-treated and the control groups

| | Grade of severity | RH* | VH* | PVR* | NV* |
|---|---|---|---|---|---|
| Octreotide | None | 56% (29) | 54% (28) | 61% (32) | 65% (34) |
| | 1 | 13% (7) | 19% (10) | 12% (6) | 10% (5) |
| | 2 | 12% (6) | 6% (3) | 19% (10) | 12% (6) |
| | 3 | 19% (10) | 21% (11) | 8% (4) | 13% (7) |
| Control | None | 54% (32) | 58% (34) | 64% (38) | 73% (43) |
| | 1 | 26% (15) | 22% (13) | 19% (11) | 14% (8) |
| | 2 | 10% (6) | 8% (5) | 15% (9) | 5% (3) |
| | 3 | 10% (6) | 12% (7) | 2% (1) | 8% (5) |

* Epiretinal hemorrhage (RH), vitreal hemorrhages (VH), proliferative vitreo-retinopathy (PVR) and neovascular budding (NV). Numbers are presented as percent of total. Absolute numbers are given in parentheses. None of the differences reached a statistical significance.

eyes. The intraobserver error for scoring of different findings was 5–10%.

Using the Spearman Rank Order Correlation test we found a strong association between the presence of PVR and epiretinal hemorrhages ($p < 0.001$) but not vitreal hemorrhages ($p = 0.9$). NV, on the other hand, was strongly associated with vitreal hemorrhages ($p < 0.001$) but not with epiretinal hemorrhages ($p = 0.27$).

The score of neovascularization-associated changes (PVR and NV) was higher in the octreotide group than in the control group (Tab. II); this difference, however, was not statistically significant. Severe (grade 3) changes were slightly more common in the octreotide-treated group (Tab. II).

### DISCUSSION

We demonstrated that twice-daily injections of octreotide failed to induce sustained inhibition of GH secretion and a reduction in serum IGF-I in neonatal rats, while pharmacological serum levels of octreotide were achieved. Furthermore, retinal neovascularization was not inhibited. If anything, severe (grade 3) manifestations of retinal neovascularization were more common in the octreotide-treated group than in the control group. These findings are in contrast with our hypothesis, based on *in vitro* studies, that octreotide prevents hypoxia-induced neovascularization either directly or by lowering GH and IGF-I levels.

The strong correlation between RH and PVR on the one hand, and between VH and NV on the other hand, is of interest and may point to some underlying pathophysiology in our model. While NV was observed mostly in the periphery of the retina (Fig. 1A), on the border of the area which is typically affected by hypoxia in the ROP model, the PVR was found mainly in the posterior pole, associated with epiretinal hemorrhages. This finding supports our theory that the proliferative vitreoretinopathy (PVR) is induced by epiretinal hemorrhage (RH), thus should not necessarily originate from the periphery of the retina, while "pure" neovascularization (NV) seen on Figure 1B is probably induced by hypoxia-related growth factors produced by adjacent avascular retina, typically in the periphery. Bleeding may either cause epiretinal proliferation directly or may be a marker of severe retinal ischemia that is triggering epiretinal proliferation through a variety of growth factors. In any case, the sub-group of eyes with intraocular hemorrhages is specifically prone to epiretinal proliferation. We should stress that our definition of neovascularization was different from that used by some other groups. We defined neovascularization as a gross histological finding of blood vessels on the inner side of internal limiting membrane, while Smith *et al.* (1997) considered every nucleus of vascular endothelial cell at the same location as evidence of neovascularization. Our way of evaluation is less sensitive, but has a greater clinical implication.

The achieved octreotide half-life of 90 minutes fits well with the previously reported half-life time of the drug (Bauer *et al.*, 1982). We evaluated the effect of octreotide in 7- and 14-day animals, since it is at this age that the avascular retina is reacting to ischemia by up-regulation of IGF-I receptor and IGF binding proteins 2 and 3 (Averbukh *et al.*, 1998). It was impossible to take blood samples from the actual experimental animals without severely affecting them by stress and anemia. On the other hand, taking blood samples at the time of sacrifice (18 days) was probably irrelevant since the course of the neovascularization process is determined much earlier.

The primary effect of octreotide on neovascularization is mediated, at least to some extent, by GH secretion inhibition. GH levels were significantly reduced two hours after injection, but returned to normal nine hours after injection. This very brief suppressive effect on GH levels is in accordance with previously published data (Wurzburger *et al.*, 1992). However, even two hours after injection, the achieved high level of octreotide failed to reduce serum IGF-I levels. It is of interest that treatment with octreotide at similar dose prevented kidney hypertrophy in diabetic rats (Flyvbjerg *et al.*, 1989) while failing to change significantly serum levels of GH or IGF-I. Smith *et al.*, showed recently that lowering of GH by another somatostatin analogue is effective in preventing hypoxia-induced neovascularization in the ROP model in mice. However, in spite of the demonstrated inhibition of GH secretion, at higher levels of somatostatin analogue there was a paradoxical relative increase in serum IGF-I levels that stayed unexplained (Smith *et al.*, 1997).

Octreotide was shown to have a direct inhibiting effect on IGF-I production in addition to its effect through the inhibition of GH secretion (Heyer *et al.*, 1989). It was previously reported that in cultured GH stimulated hepatocytes, IGF-I mRNA levels were significantly reduced in the presence of low octreotide concentrations (0.3 and 3 μg/l) while high concentrations of octreotide (30 and 300 μg/l) had no effect on IGF-I mRNA abundance (Serri *et al.*, 1992). In the present study, octreotide was injected at 12-hour intervals; therefore the lowest serum level of octreotide, just before the next injection, was approximately 6.6 μg/l (extrapolated) which may be still too high for a direct suppressive effect on IGF-I production but too low for suppression of GH secretion. Moreover, withdrawal of octreotide, like somatostatin withdrawal, may generate a pulsatile GH release (Cella *et al.*, 1996) which may explain the elevation of IGF-I levels at 9 and 14 hours after injection. Fluctuations in IGF-I levels, secondary to rebound elevation of GH after a brief suppression, may have a deleterious effect on retinal vasculature, promoting rather than suppressing neovascularization.

Finally, octreotide was found to have direct antiangiogenic properties in human retinal endothelial cell (HREC) culture (Grant *et al.*, 1993b) and in a vascular growth model of the chick-embryo chorioallantoic membrane (Woltering *et al.*, 1991). In cell culture of HREC stimulated by IGF-I and bFGF, [$^3$H]thymidine incorporation was inhibited by octreotide (Grant *et al.*, 1993b). The dose–effect curve showed that an octreotide level of 1.6 μg/l caused 15% inhibition, 16 μg/l caused 35% inhibition and 164 μg/l caused 60% inhibition. In our experiment, octreotide serum level was between a few μg/l and a few hundreds μg/l during the whole 12-hour interval between injections. Inhibition of angiogenesis, however, was not achieved.

In conclusion, twice-daily injections of octreotide did not prevent hypoxia-related neovascularization in our model. It is possible, however, that the therapeutic range of octreotide is of critical importance, and that the great variability of octreotide levels achieved in our study due to twice-daily injections is not suitable for a sustained effect. Furthermore, fluctuations in serum octreotide level may have caused the observed increase in neovascularization-related changes in the treated group. Continuous infusion of octreotide and other somatostatin analogues with a longer half-life that better suppress GH secretion may still be effective in preventing neovascularization.

### *Acknowledgments*

Supported by a grant from the Pauline and Joseph Fried Family, and by a grant from the Chief Scientist, Ministry of Health, Israel.

### *References*

Averbukh, E., Weiss, O., Halpert, M., Yanko, R., Moshe, R., Nephesh, I., Flyvbjerg, A., Yanko, L. and Raz, I. (1998) Gene expression of IGF-I, its receptor and binding proteins in retina under hypoxic conditions. *Metabolism*, **47**, 1331–1336.

Bauer, W., Briner, U., Doepfner, W., Haller, R., Huguenin, R., Marbach, P., Petcher, T. J. and Pless, J. (1982) SMS 201-995: a very potent and selective octapeptide analogue of somatostatin with prolonged action. *Life Sci.*, **31**, 1133–1140.

Bradley, R. F., Rees, S. B. and Fager, S. B. (1965) Pituitary ablation in the treatment of diabetic retinopathy. *Med. Clin. North Am.*, **49**, 1105–1124.

Cella, S. G., Luceri, M., Cattaneo, L., Torsello, A. and Muller, E. E. (1996) Somatostatin withdrawal as generator of pulsatile GH release in the dog: a possible tool to evaluate the endogenous GHRH tone? *Neuroendocrinol.*, **63**, 481–488.

Chang-Ling, T., Tour, S., Hollander, H. and Stone, J. (1992) Vascular changes and their mechanisms in the feline model of retinopathy of prematurity. *Invest. Ophthalmol. Vis. Sci.*, **33**, 2128–2147.

Dills, D. G., Moss, S. E., Klein, R. and Klein, B. E. K. (1991) Association of elevated IGF-I levels with increased retinopathy in late-onset diabetes. *Diabetes*, **40**, 1725–1730.

Flyvbjerg, A., Frystyk, J., Thorlacius-Ussing, O. and Ørskov, H. (1989) Somatostatin analogue administration prevents increase in kidney somatomedin C and initial renal growth in diabetic and uninephrectomized rats. *Diabetologia*, **32**, 261–265.

Furlanetto, R. W. and Marino, J. M. (1987) Radioimmunoassay of somatomedin C/insulin-like growth factor I. In: Colowick, S. P. (Ed.) *Methods in Enzymology: Peptide growth factors, part A* (New York: Academic Press), **146**, 216–226.

Grant, M. B., Mamer, R. N., Fitzgerald, C., Ellis, E. A., Aboufriekha, M. and Guy, J. (1993) Insulin-like growth factor I acts as an angiogenic agent in rabbit cornea and retina: comparative studies with basic fibroblast growth factor. *Diabetologia*, **36**, 282–291.

Grant, M. B., Caballero, S. and Millard, W. J. (1993) Inhibition of IGF-I and b-FGF stimulated growth of human retinal endothelial cells by the somatostatin analogue, octreotide: a potential treatment for ocular neovascularization. *Regul. Pept.*, **48**, 267–278.

Heyer, S. L., Sharp, P. S., Brooks, R. A., Burrin, J. M. and Kohner, E. M. (1989) Continuous subcutaneous octreotide infusion markedly suppresses IGF-I levels whilst only partially suppressing GH secretion in diabetics with retinopathy. *Acta Endocrinol.*, **120**, 187–194.

Kierkegaard, C., Nørgaard, K., Snorgaard, O., Bek, T., Larsen, M. and Lund-Andersen, H. (1990) Effect of one year continuous subcutaneous infusion of a somatostatin analogue, octreotide, on early retinopathy, metabolic control and thyroid function in Type I (insulin-dependent) diabetes mellitus. *Acta Endocrinol.*, **122**, 766–772.

Kohner, E. M., Joplin, J. F., Cheng, H., Blach, R. K. and Fraser, T. R. (1972) Pituitary ablation in treatment of diabetic retinopathy. *Trans. Ophthalmol Soc. UK*, **92**, 79–90.

Lee, H. K., Suh, K. I., Koh, C. S., Min, H. K., Lee, J. H. and Chung, H. (1988) Effect of SMS 201-995 in rapidly progressive diabetic retinopathy. *Diabetes Care*, **11**, 441–442.

Lundbaek, K., Christensen, N. J., Jensen, V. A., Johansen, K., Olsen, T. S., Hansen, A. P., Ørskov, H. and Osterby, R. (1970) Diabetes, diabetic angiopathy and growth hormone (hypothesis). *Lancet*, **2**, 131–133.

Mallet, B., Vialettes, B., Haroche, S., Escoffier, P., Gastaut, P., Taubert, J. P. and Vague, P. (1992) Stabilization of severe proliferative diabetic retinopathy by long-term treatment with SMS 201-995. *Diabetes Metab.*, **18**, 438–444.

McCombe, M., Lightman, S., Eckland, D. J., Hamilton, A. M. and Lightman, S. L. (1991) Effect of a long-acting somatostatin analogue (BIM23014) on proliferative diabetic retinopathy: pilot study. *Eye*, **5**, 569–575.

Merimee, T. J., Zapf, J. and Froesch, E. R. (1983) Insulin-like growth factors: studies in diabetics with and without retinopathy. *N. Eng. J. Med.*, **309**, 527–530.

Meyer-Schwickerath, R., Pfeiffer, A., Blum, W. F., Freyberger, H., Klein, M., Losche, C., Rollmann, R. and Schatz, H. (1993) Vitreous levels of the insulin-like growth factors I and II, and the insulin-like growth factor binding proteins 2 and 3, increase in neovascular eye disease. Studies in nondiabetic and diabetic subjects. *J. Clin. Invest.*, **96**, 2620–2625.

Ørskov, H., Thomsen, H. G. and Yde, H. (1968) Wick chromatography for rapid and reliable immunoassay of insulin, glucagon and growth hormone. *Nature*, **219**, 193–195.

Penn, J. S., Tolman, B. L. and Lowery, L. A. (1993) Variable oxygen exposure causes preretinal neovascularization in newborn rat. *Invest. Ophthalmol. Vis. Sci.*, **34**, 576–585.

Pierce, E. A., Foley, E. D. and Smith, L. E. (1996) Regulation of vascular endothelial growth factor by oxygen in a model of retinopathy of prematurity. *Arch. Ophthalmol.*, **114**, 1219–1228.

Plewe, G., Noelken, G., Krause, U., Beyer, J. and del Poso, E. (1987) Suppression of growth hormone and somatomedin C by long-acting somatomedin analogue SMS 201-995 in Type I diabetes mellitus. *Hormone Res.*, **27**, 7–12.

Reynaud, X. and Dorey, C. K. (1994) Extraretinal neovascularization induced by hypoxic episodes in the neonatal rat. *Invest. Ophthalmol. Vis. Sci.*, **35**, 3169–3177.

Serri, O., Brazeau, P., Kachra, Z. and Posner, B. (1992) Octreotide inhibits insulin-like growth factor-I hepatic gene expression in the hypophysectomized rat: evidence for a direct and indirect mechanism of action. *Endocrinology*, **130**, 1816–1821.

Smith, L. E. H., Kopchick, J. J., Chen, W., Knapp, J., Kinose, F., Daley, D., Foley, E., Smith, R. G. and Schaeffer, J. M. (1997) Essential role of growth hormone in ischemia-induced retinal neovascularization. *Science*, **276**, 1706–1709.

Woltering, E. A., Barrie, R., O'Dorisio, T. M., Arce, D., Ure, T., Cramer, A., Holmes, D., Robertson, J. and Fassler, J. (1991) Somatostatin analogues inhibit angiogenesis in the chick chorioallantoic membrane. *J. Surg. Res.*, **50**, 245–251.

Wurzburger, M. I., Prelevic, G. M., Sönksen, P. H. and Balint-Peric, L. A. (1992) The effect of the somatostatin analogue octreotide on growth hormone secretion in insulin-dependent diabetics without residual insulin secretion. *Horm. Metab. Res.*, **24**, 329–332.

